# Exercise Does Not Protect against Peripheral and Central Effects of a High Cholesterol Diet Given *Ad libitum* in Old ApoE^−/−^ Mice

**DOI:** 10.3389/fphys.2016.00453

**Published:** 2016-10-06

**Authors:** Vanessa Di Cataldo, Alain Géloën, Jean-Baptiste Langlois, Fabien Chauveau, Benoît Thézé, Violaine Hubert, Marlène Wiart, Erica N. Chirico, Jennifer Rieusset, Hubert Vidal, Vincent Pialoux, Emmanuelle Canet-Soulas

**Affiliations:** ^1^Univ Lyon, CarMeN Laboratory, Institut National de la Santé et de la Recherche Médicale U1060, INRA U1397, Université Lyon 1, INSA Lyon, F-69600Oullins, France; ^2^CERMEP—Imagerie Du VivantLyon, France; ^3^Lyon Neuroscience Research Center, Centre National de la Recherche Scientifique UMR5292, Institut National de la Santé et de la Recherche Médicale, University Lyon 1Lyon, France; ^4^Laboratoire Imagerie Moléculaire In vivo, UMR 1023 Institut National de la Santé et de la Recherche Médicale /CEA/Université Paris Sud - ERL 9218 Centre National de la Recherche Scientifique, CEA/I^2^BM/SHFJOrsay, France; ^5^Centre National de la Recherche Scientifique UMR 5220, Institut National de la Santé et de la Recherche Médicale U.1060 (CREATIS), University Claude Bernard Lyon1INSA de Lyon, France; ^6^University of Lyon, University Lyon 1, Laboratoire Inter-Universitaire de Biologie de la Motricité (EA647)Villeurbanne, France

**Keywords:** atherosclerosis, neuroinflammation imaging, overfeeding, oxidative stress, physical activity

## Abstract

**Aim:** Advanced atherosclerosis increases inflammation and stroke risk in the cerebral vasculature. Exercise is known to improve cardio-metabolic profiles when associated with a caloric restriction, but it remains debated whether it is still beneficial without the dietary control. The aim of this study was to determine both the peripheral and central effects of exercise training combined with a cholesterol-rich diet given *ad libitum* in old ApoE^−/−^ mice.

**Methods**: Forty-five-weeks old obese ApoE^−/−^ mice fed with a high cholesterol diet *ad libitum* were divided into Exercise-trained (EX; running wheel free access) and Sedentary (SED) groups. Insulin tolerance and brain imaging were performed before and after the twelve-weeks training. Tissue insulin resistance, oxidative stress, and inflammation markers in plasma, aorta, and brain were then assessed.

**Results**: In EX ApoE^−/−^ mice, no beneficial effect of exercise was observed on weight, abdominal fat, metabolic parameters, oxidative stress, or inflammation compared to SED. Despite the regular exercise training in ApoE^−/−^ EX mice (mean of 12.5 km/week during 12 weeks), brain inflammation imaging score was significantly associated with increased blood brain barrier (BBB) leakage evaluated by imaging follow-up (*r*^2^ = 0.87; *p* = 0.049) with a faster evolution compared to SED ApoE^−/−^mice.

**Conclusion**: We conclude that in a context of high cardio-metabolic risk, exercise does not provide any protective effect in old ApoE^−/−^ animals under high cholesterol diet given *ad libitum*. Peripheral (insulin sensitivity and oxidative/inflammatory status) but also central features (BBB preservation and protection against inflammation) did not show any benefits of exercise. Indeed, there was a fast induction of irreversible brain damage that was more pronounced in exercise-trained ApoE^−/−^ mice.

## Introduction

Numerous studies have shown that regular physical activity had protective effects against chronic diseases, like in atherosclerosis (Szostak and Laurant, 1979; Laufs et al., 2005) by decreasing oxidative stress and inflammation. Through these protective effects, it is suggested that regular exercise training could decrease the risks of developing cardio and cerebrovascular complications (Pialoux et al., 2009). Overall, exercise training may create a favorable anti-inflammatory and antioxidant environment, counter-acting both local inflammatory burst, and systemic metabolic low-grade inflammation (Pialoux et al., 2009; Gleeson et al., 2011).

Central effects of exercise are less explored in the context of atherosclerosis. Advanced atherosclerosis increases the risk of stroke, inflammation, and oxidative stress in the cerebral vasculature. Aged ApoE^−/−^ mice have a compromised blood-brain barrier (BBB), with an increased susceptibility to ischemic damage which is further altered by the high fat/high cholesterol diet (Hafezi-Moghadam et al., 2006; ElAli et al., 2011; Badaut et al., 2012). In addition to the vascular risk *per-se*, an increasing number of studies are reporting various locations of BBB permeability and hippocampal inflammation as a direct consequences of obesity and high fat consumption (Badaut et al., 2012; Thaler et al., 2012; Lee et al., 2013; Erion et al., 2014; Van der Donckt et al., 2015). In an atherosclerosis mouse model under high fat diet, we and others have recently shown that exercise can limit brain inflammation (Yi et al., 2012; Auer et al., 2015; Chirico et al., 2016).

On the other hand, whereas systemic benefits of physical activity on the cardio-metabolic profile are commonly recognized, there is a significant proportion of non-responders, showing no or even adverse exercise effect on glucose homeostasis in large clinical studies (Böhm et al., 2016). In some studies, it was found to be highly dependent on food intake and its effect on body weight (Bergouignan et al., 1985). Indeed, in obese subjects who had the same physical activity level, it was observed that those under food restriction presented reduced oxidative stress, inflammation, and insulin resistance compared to those with no food restriction and increased body weight (Bergouignan et al., 1985). This interplay between food intake and the peripheral response to physical training may also impact the central effects of training (Hicks et al., 2016), but has not been tested yet in a context of high cardiovascular risk.

Imaging biomarkers are increasingly used to evaluate progression of brain abnormalities. BBB damage is currently measured *in vivo* by the extent of gadolinium leakage in T1-weighted magnetic resonance imaging (MRI; Nighoghossian et al., 2007). Under conditions of acute or chronic inflammation, macrophages infiltrate the cerebral parenchyma, and exhibit an important phagocytic activity, that can be seen by ultrasmall superparamagnetic particles of iron oxide (USPIOs) enhanced MRI in stroke patients (Nighoghossian et al., 2007). In the cerebral parenchyma, several publications have reported that microbleeds (Akoudad et al., 2014) and brain accumulation of erythrocytes and iron (Schreiber et al., 2012) can also be detected *in vivo* using T2 and T2^*^ MRI (Wardlaw et al., 2013).

Using imaging and insulin tolerance test for follow-up, the aim of the present study was to determine both the peripheral and central effects of exercise training in 45 weeks-old ApoE^−/−^ mice fed *ad libitum* with a high fat/ high cholesterol (HC) diet.

## Materials and methods

### Animals and training

**Figure 1:**
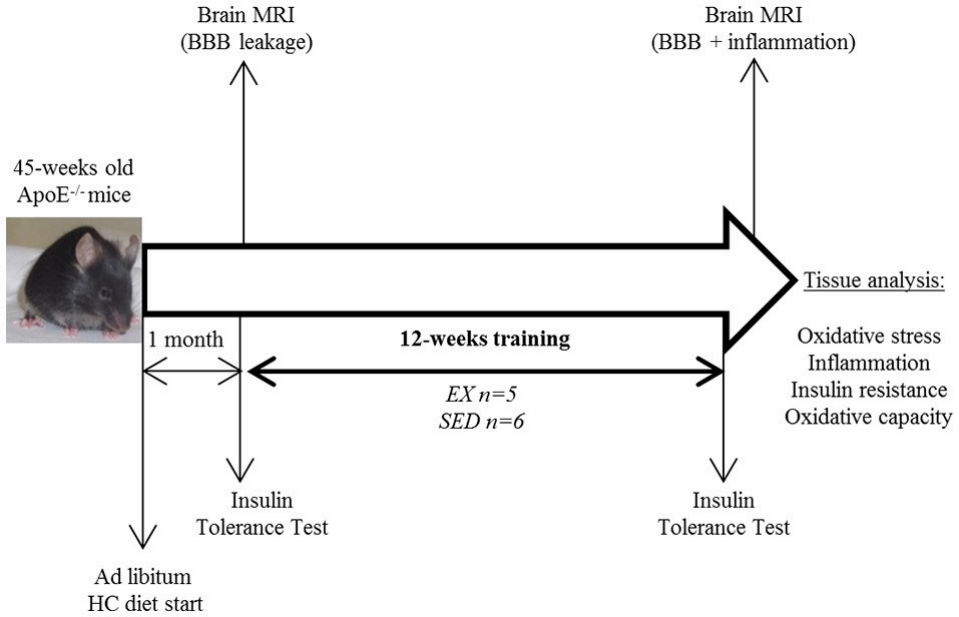
**Study design**. HC diet, high cholesterol diet; BBB, blood-brain-barrier; EX, exercise trained mice; SED, sedentary mice.

All procedures were in conformity with the European regulation for animal use and this study was approved by the local ethics committee of the institution (Comité d'éthique de l'INSA de Lyon Cetil *n*°102). ApoE^−/−^ mice (C57Bl/6 background, Charles River, France *n* = 14) were fed a high cholesterol (HC) diet (Safe U8220 v.153: A04 + 21% fat, 0.15% cholesterol, SAFE, Augy, France) starting 1 month before the training and until the end of the study. Animals were males and females equally assigned in both groups and maintained on a 12 h light-dark cycle and were supplied with food and water *ad libitum*. After careful maintenance of health conditions during 1 year (Guerbet, Animal Care Unit, France), 45 weeks-old mice were randomly divided into 2 activity groups. Mice in the exercise-trained (EX) group individually housed in cages equipped with a 12.5 cm metal running wheel (HAGEN-61700, Montreal, Canada; Goh and Ladiges, 2015) and digital magnetic counter (model BC906 Sigma Sport, Neustadt, Germany), while the sedentary (SED) group had a standard cage (Figure 1). Sedentary mice were caged by 3–4 in order to avoid excessive stress due to loneliness (and not compensated by the possibility to exercise contrary to trained mice). During the 12 weeks of training, the distance ran and the general health of the mice were recorded one time a week. A regular check-up of mice was performed (twice a week) and the exclusion criterion was overall bad health of the animal (i.e., tumors, hemiparesis, large skin irritations). Before sacrifice for blood and tissue assessment, both exercise and sedentary mice were caged by 3–4, with no wheel access, and fasted for 12 h.

Wild type (WT) C57Bl/6 mice of matched age fed with a standard diet (12% fat, Teklad Global 16% protein, HARLAN, Gannat, France) and divided into EX and SED groups were also studied to provide reference values of metabolic and inflammatory markers in normal animals not at risk of vascular lesions.

### Intraperitoneal insulin tolerance test (ITT)

Glycaemia was measured over 45 min in 6 h-fasted mice using a glucometer (AccuCheck, Roche, Germany) after an initial tail puncture, corresponding to baseline glycaemia. Immediately after, all mice were sequentially injected by insulin (intraperitoneal injection, 0.75mU.g^−1^ of body weight). Fifteen minutes after insulin injection, glycaemia was assessed following the same sequence, and measurements were repeated at 30 and 45 min. ITT were performed before and after training, prior to MRI sessions. Insulin response was determined by area under curve (AUC).

### Brain MRI

For the imaging protocol, mice were anesthetized by isoflurane (4% for induction, 1–2% for maintenance; TEM SEGA, Pessac, France). The scanning support bed used for the experiment was equipped with a warm water recirculation system and a respiratory sensor, which monitored the respiration rate throughout the scan. MRI acquisitions were performed on a Bruker BioSpec 7T system (Bruker Biospin, Ettlingen, Germany).

For brain imaging, a Bruker birdcage volume coil (outer diameter = 112 mm and inner diameter = 72 mm) was used for the signal transmission, and a Bruker single loop surface coil (15 mm diameter) positioned over the head was used for signal reception. Brain T2-weighted spin-echo images were acquired using a rapid acquisition with relaxation enhancement (RARE) sequence on axial plane. To further characterize the neurovascular lesions, an extended brain MRI protocol was performed (see supplemental methods in Supplementary Material). Vascular lesions and hemorrhage/erythrocytes/iron accumulation were assessed respectively by baseline T2 and T2^*^ imaging (Schreiber et al., 2012; Akoudad et al., 2014). T2^*^ mapping was obtained using a multi-slice multi-echo gradient echo sequence. Gadolinium chelate (Gd-DOTA, 0.1 mmol Gd.kg^−1^, Guerbet, Aulnay-sous-Bois, France) was then retro-orbitally injected to observe possible BBB leakage. Macrophage imaging was performed using USPIO injection (P904, 1mmol Fe.kg^−1^, Guerbet, Aulnay-sous-Bois, France) and the 48 h post-contrast T2/T2^*^ for inflammation imaging, as previously described (Sigovan et al., 2012). The total duration of the MRI protocol was less than 2 h.

In addition, to assess body composition in both groups, whole body fat evaluation was performed in representative individuals of each group using 2D water/fat gradient echo acquisition and whole body nuclear magnetic resonance (NMR) spectrum at the end of the after-training MRI session (Naville et al., 2015). Results are presented as relative fat over water after analysis of corresponding peaks in the MR spectrum.

### MRI analysis

For brain analysis, pre-contrast T2/T2^*^ images and post-gadolinium images were categorized based on the size of abnormal areas and the number of slices affected. Two observers scored anonymized data obtained before and after training, i.e., pre- and post- contrast (48 h post-P904, after-training MRI session) on T2/T2^*^ images and gadolinium leakage on post-gadolinium T1-weighted images. Briefly, a score of 1–4 was given for abnormalities seen on pre-contrast images and a score of 1–4 was given for changes seen between pre and post-contrast images. A score of 1 is given when no abnormality was observed, 2 when small lesions (< 10 pixels) appeared on one to two slices, 3 for medium size lesion (10 < pixels < 20) on one to two slices and finally a score of 4 represents a large lesion (>20 pixels) on at least two slices. In order to evaluate the evolution of BBB leakage, the delta of T1 (ΔT1) score was calculated by subtracting post-training to pre-training score.

### Dissection

Following the second imaging session, mice were anesthetized by intraperitoneal injection of pentobarbital (50 mg.kg^−1^, Dolethal®, Vétoquinol, Lure, France) and blood was collected by cardiac puncture. The heart was transcardially perfused for 70 s with 9% NaCl. The brain, ascending and abdominal aorta, gastrocnemius, soleus, and visceral and subcutaneous adipose tissue were removed. Sections to be used for biological assays were stored at −80°C until assessment.

### *Ex vivo* insulin signalization test

As previously described (Rieusset et al., 2012), immediately after sampling, gastrocnemius muscles were finely cut and incubated for 15 min at 37°C in 3 ml of low glucose Dulbecco's Modified Eagle's Medium (DMEM 1g.L^−1^) for the sample (−) and low glucose DMEM + 5% insulin 10^−7^mol for the sample (+) and then quickly wiped and stored in liquid nitrogen. Samples (−) were used to study the expression of basal protein kinase B (PKB) and phosphorylated PKB (pPKB), while samples (+) to measure the quality of insulin signaling. Samples were kept at −80°C. Proteins of these muscles were then extracted by grinding with a Polytron in RIPA+ and centrifuged for 10 min at 3750 rpm at 4°C. The supernatant were recovered and diluted 1:5 in water in order to be assayed by the Bradford method. Then proteins (30 μg) were loaded on a precast gel (CRITERION TGX Stain-Free 10% acrylamide, BioRad, Hercules, CA, USA) to study muscle pPKB (#4060, Cell signaling, Danvers, MA, USA) and PKB (#9272, Cell signaling, Danvers, MA, USA) expression by Western Blot, and its response to insulin stimulation. Membranes are PVDF from BioRad Company (BioRad, Hercules, CA, USA).

### Sample preparation in liquid nitrogen

Tissue samples were stored at −80°C. Each one was weighed then put in a mortar placed in a polystyrene box filled with liquid nitrogen to be grounded into a powder. The powders obtained are divided into three equal parts of 80–120 mg, distributed separately to study ribonucleic acid (RNA), proteins and oxidative stress, and stored at −80°C.

### Biological analysis:

All tissues were kept frozen and homogenized with a 10% v/w buffer (PBS + 0.5mM EDTA). Homogenates were centrifuged at 4°C for 4 min at 1500 g for protein content and malondialdehyde (MDA) analysis, and again at 4°C for 10 min at 12,000 g for the remaining analyses. Supernatants were frozen at −80°C. Protein concentrations were determined spectrophotometrically (Biophotometer, Eppendorf, Germany) using a bicinchoninic acid (BCA) kit according to instructions (Sigma, St Louis, USA).

### Citrate synthase

Muscle adaptation to physical activity was determined by skeletal muscle citrate synthase activity measuring using soleus muscle homogenate according to the Shepherd and Garland method (Shepherd and Garland, 1969).

### Cholesterol assay

Total blood cholesterol was assessed using Amplex Red Cholesterol Assay Kit (Invitrogen, Carlsbad, CA) following manufacturer instructions.

### Oxidative stress

Oxidative stress markers (Advanced Oxidation Protein Products, AOPP and Malondialdehyde, MDA) and antioxidants markers (Superoxide dismutase, SOD and Glutathione peroxidase, GPx) were measured in plasma and brain as previously described (Chirico et al., 2012) using the method of Witko-Sarsat et al for AOPP (Witko-Sarsat et al., 1996), Ohkawa et al for MDA (Ohkawa et al., 1979), Oberley and Spitz for SOD (Oberley and Spitz, 1984), and Paglia and Valentine for GPx (Paglia and Valentine, 1967). All reagents used for biochemical assays were purchased from Sigma Aldrich. AOPP and SOD were also measured in descending aorta of ApoE^−/−^ mice.

### Inflammatory markers

Inflammation status was assessed in plasma, brain, and descending aorta supernatant using a commercially available mouse enzyme-linked immunosorbent assay kit (IL-1β: ELM-IL1β-001, Raybiotech; TNFα: Mouse TNF BD OptEIA Kit, BD Biosciences) according to manufacturer's instructions.

### Immunochemistry

Brain samples were harvested and fixed in a 4% paraformaldehyde solution during 1 h followed by sucrose for 24 h and preserved at −80°C until processing. Three successive 15 μm-thick sections for 3 MRI locations were assessed with DeadEnd™ Fluorometric TUNEL System kit (PROMEGA, San Luis Obispo, CA, USA) to detect DNA fragmentation (apoptosis), rat anti F4/80 antibody (MF48000, 1/200, CALTAG MEDSYSTEMS, Buckingham, UK) to detect macrophages and AlexaFluor 594 nm (AF594) goat anti-mouse IgG (A11032, Life Technologies, USA) for blood brain barrier (BBB) permeability. AF594 goat anti rat IgG (A11007, 1/1000, Life Technologies, USA) was used as secondary antibody for F4/80 labeling. Slides were mounted with Prolong gold anti-fade reagent (P36935, Life Technologies, USA) supplemented with DAPI for nuclear counterstain. Image acquisition was performed with an Axio Observer Z1 Zeiss microscope.

### Statistics

Analyses were conducted using Statistica (version 8.0, Statsoft, Tulsa, OK, USA). As our study is based on longitudinal imaging features, in-house power analysis for assessing the brain effect of HC diet in ApoE^−/−^ mice has shown that 6–7 mice per group are sufficient to reach the statistical potency with 80–90% power. Results are presented as mean ± standard error of the mean (SEM). A normality test (Kolgomorov-Smirnov test) has been applied and the distribution of our data is non-parametric. Statistical comparisons between ApoE^−/−^SED vs. EX were performed by Mann-Whitney U test and comparisons between before and after-training were performed by Wilcoxon test. Linear regression was used for MR scores comparison and Spearman correlation coefficient was measured. Statistical significance was determined by a *p* < 0.05.

## Results

**Figure 2:**
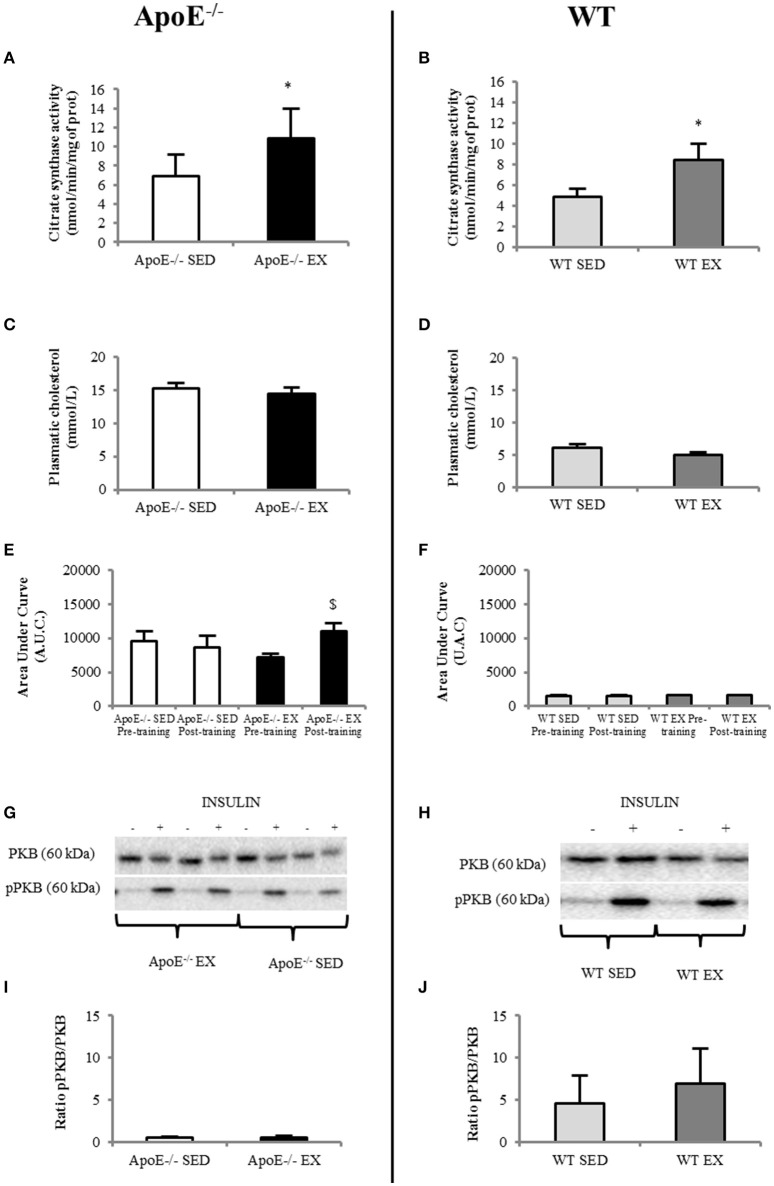
**In EX (*n* = 5)ApoE^−/−^ mice, citrate synthase activity in the soleus muscle increased significantly compared to SED mice (*n* = 6) (A) whereas plasmatic cholesterol level was similar (C); systemic insulin resistance (comparison of before and after-training insulin tolerance test, ITT, showed a significant degradation in EX ApoE^−/−^ mice (E) (*p* = 0.021 on before vs. after-training ITT in EX ApoE^−/−^)**. Insulin signalization in gastrocnemius muscle was impaired in both SED and EX mice, as shown by pPKB/PKB Western Blot **(G)** and ratio **(I)**. WT mice values are given for reference (SED = 7; EX = 6) for citrate synthase activity in the soleus muscle **(B)** significantly increased in Ex compared to SED mice), plasmatic cholesterol level **(D)**, ITT **(F)**, pPKB/PKB Western Blot **(H)**, and ratio **(J)**. Significantly different in EX compared to SED: ^*^p < 0.05; significantly different before vs. after: ^$^*p* < 0.05.

Three ApoE^−/−^ mice on HC diet (1 SED and 2 EX) died during the training period. For the 11 animals (5 EX and 6 SED) that completed the entire protocol, weekly running distance and body weight before and after training are detailed in Table 1. Oxidative enzyme citrate synthase level in the soleus were higher in EX than in SED ApoE^−/−^mice (*p* = 0.042; Figure 2A). A similar EX to SED differences is observed in the aged-matched WT mice (Figure 2B), supporting the physiological efficiency of the exercise training.

**Table 1:** **Training effect: running distances and weight of ApoE^−/−^(SED = 6; EX = 5) and WT mice (for reference values; SED = 7; EX = 6)**.

	**Group**	**ApoE^−/−^**		**WT**	
		**SED**	**EX**	**SED**	**EX**
Before training	Weight (g)	44.7 ± 6.6	40.6 ± 2.5	27.3 ± 0.8	27.4 ± 0.8
After training	Weight (g)	44.3 ± 6.3	45 ± 3.1^*^	27.2 ± 0.5	27.7 ± 0.4
	MeanΔweight (%)	1%	18%	< 1%	< 1%
	Running distances (km/week)	N/A	12.5 ± 5.4	N/A	16.2 ± 3.6

* p < 0.05 Significantly different from before training.

Despite a substantial running distance, EX ApoE^−/−^ significantly increased their body weight (+18%, *p* = 0.027) during the 12-weeks training (Table 1). Moreover, both EX and SED ApoE^−/−^ mice were already obese at the beginning of the study (Table 1, Figure S1).The fat index measured using MR spectroscopy showed no difference of fat content, and 3D fat MRI, no apparent difference of fat distribution in ApoE^−/−^EX mice compared to their sedentary counterparts (Figure S1). Furthermore, visceral and subcutaneous adipose tissues had similar weight in both EX and SED ApoE^−/−^ mice (Table S1).

### Metabolic parameters

Plasma cholesterol was high in ApoE^−/−^ mice and was not modified by exercise training (i.e., no difference between EX and SED ApoE^−/−^, Figure 2C). It was normal in both EX and SED WT mice, and not modified by exercise training (Figure 2D). Insulin tolerance test showed low insulin tolerance in ApoE^−/−^ mice independently of training paradigm and time of the study as demonstrated by the large Area Under Curve of glucose concentration over time (AUC, Figure 2E). WT values are given in Figure 2F. It should also be pointed that ApoE^−/−^ EX mice significantly further lowered their insulin tolerance after the training (*p* = 0.027) (Figures 2C and Figure S2).

Western blots of pPKB/PKB in gastrocnemius confirmed that both EX and SED ApoE^−/−^ mice had muscle insulin resistance (Figures 2G,I).Values of aged-matched EX and SED WT mice are presented in Figures 2H,J. There was no beneficial effect of physical activity on muscle insulin sensitivity in ApoE^−/−^ mice (Figure 2I).

### Brain imaging and immunochemistry

**Figure 3:**
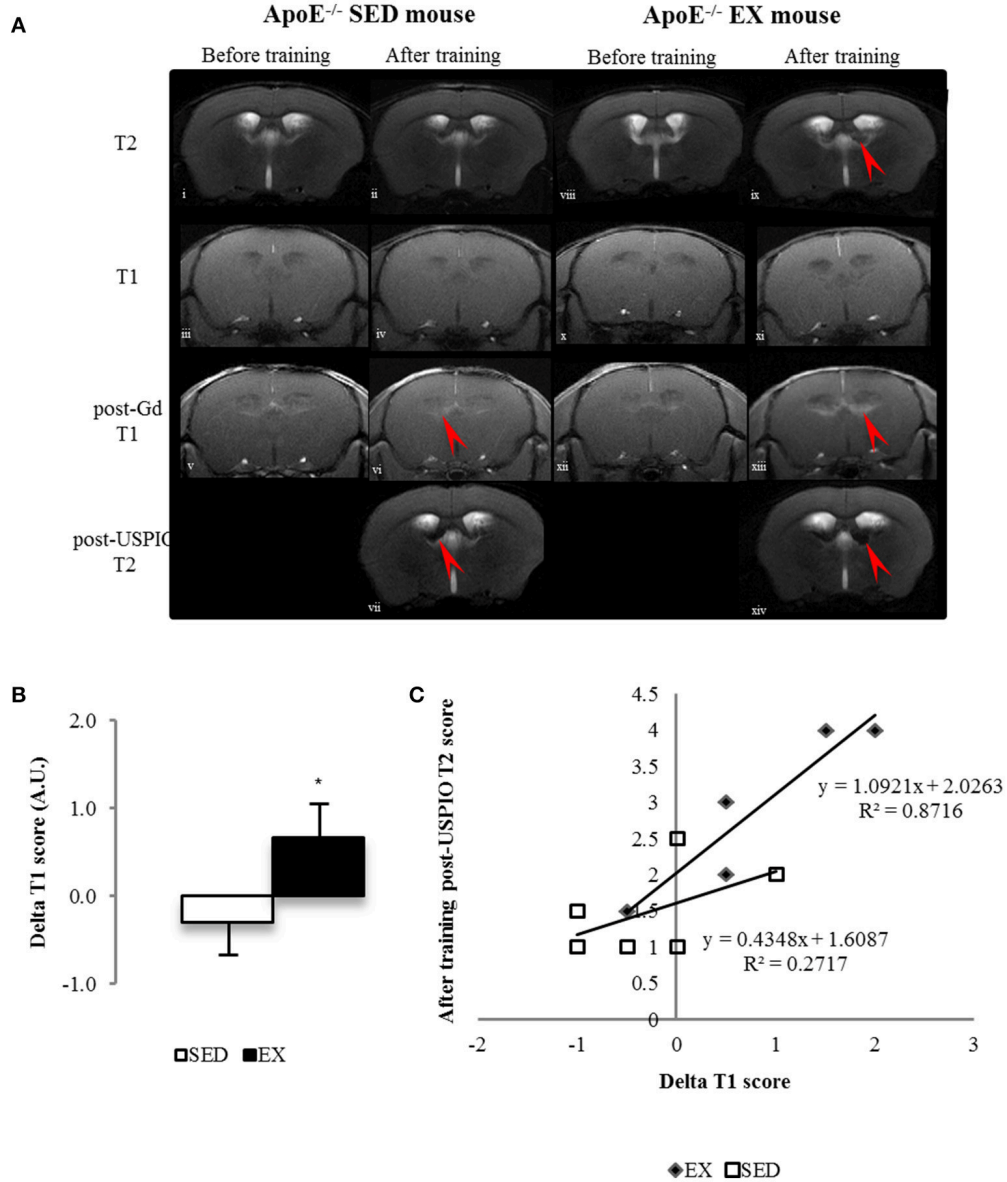
**Longitudinal brain MR images of a SED (i–vii) and an EX ApoE^−/−^ mouse (viii–xiv)**. before (i, viii) and after training (ii, ix) native T2 image; native (iii, iv, x, xi) and Post-Gadolinium (Gd) T1 images (v, vi, xii, xiii), the enhancing bright zone after Gd showing BBB leakage in ApoE^−/−^ mouse (vi, red arrow); Post-USPIO (vii, xiv) T2 images, the loss of signal showing USPIO phagocytosis by macrophages in ApoE^−/−^ (vii, red arrow) **(A)** Delta score of longitudinal brain MR Imaging before and after-training score of post-Gd T1 **(B)** in SED and EX ApoE^−/−^ mice **(B)** (*p* = 0.02 compared to SED). Linear regression of Delta T1 (ΔT1) scores and after-training post-USPIOs T2 scores of EX and SED ApoE^−/−^ mice showed a stronger correlation between increased BBB leakage and high phagocytic activity in EX mice **(C)** (*p* = 0.049). Significantly different from SED mice: ^*^p < 0.05.

Brain MRI performed at the beginning of the study showed that ApoE^−/−^ mice brain have focal areas (periventricular area) of BBB leakage on post-gadolinium T1 images (Figure 3).

Analysis of longitudinal data showed that these features, present in old ApoE^−/−^ mice (Chirico et al., 2016), evolved during the 12-weeks training in EX ApoE^−/−^ mice, as confirmed by the significant increase in post-gadolinium T1 score (*p* = 0.02, Figure 3A–B), when there was no significant change in SED ApoE^−/−^ mice. After-training, focal periventricular areas of signal loss were observed on T2^*^ maps in both EX and SED ApoE^−/−^ mice (Figure S3), but there were no significant T2 and T2^*^ scores changes after the follow-up period. After-training post-USPIOs score showed more extended inflammation areas in EX ApoE^−/−^ mice compared to SED (*p* = 0.015, Table 2 and Figure S3). Inflammation areas co-localized with the periventricular BBB leakage and abnormalities on T2/T2^*^ images (Figure 3 and Figure S3). Post-USPIOs T2 score was significantly associated with the increased BBB leakage (ΔT1 score) in EX ApoE^−/−^ mice (*p* = 0.049, Figure 3C).

**Table 2:** After—training comparison of brain MR imaging scores of ApoE^−/−^: vascular lesions (T2^*^ score), USPIO's assessment of inflammation (post-USPIOs T2^*^ score) and post-Gd BBB leakage (post-gadolinium T1-weighted sequence).

	**ApoE^−/−^ SED**	**ApoE^−/−^ EX**
T2^*^ score	1.4 ± 0.3 (2)	1.2 ± 0.1 (4)
Post-USPIOs T2^*^ score	1.6 ± 0.3 (2)	2.6 ± 1.0.5 (5)^*^
Post-Gd T1 score	2.4 ± 0.5 (4)	2.3 ± 0.4 (5)

In brackets, number of mice with score >1.

* p < 0.05 Significantly different from ApoE^−/−^ SED mice (mean ± SEM).

There were no such features and no changes during follow-up in both EX and SED WT mice (data not shown, mean score of one for T1, T2, and T2^*^ before and after training), and no periventricular post-USPIOs abnormalities on T2^*^ maps (Figure S3).

**Figure 4:**
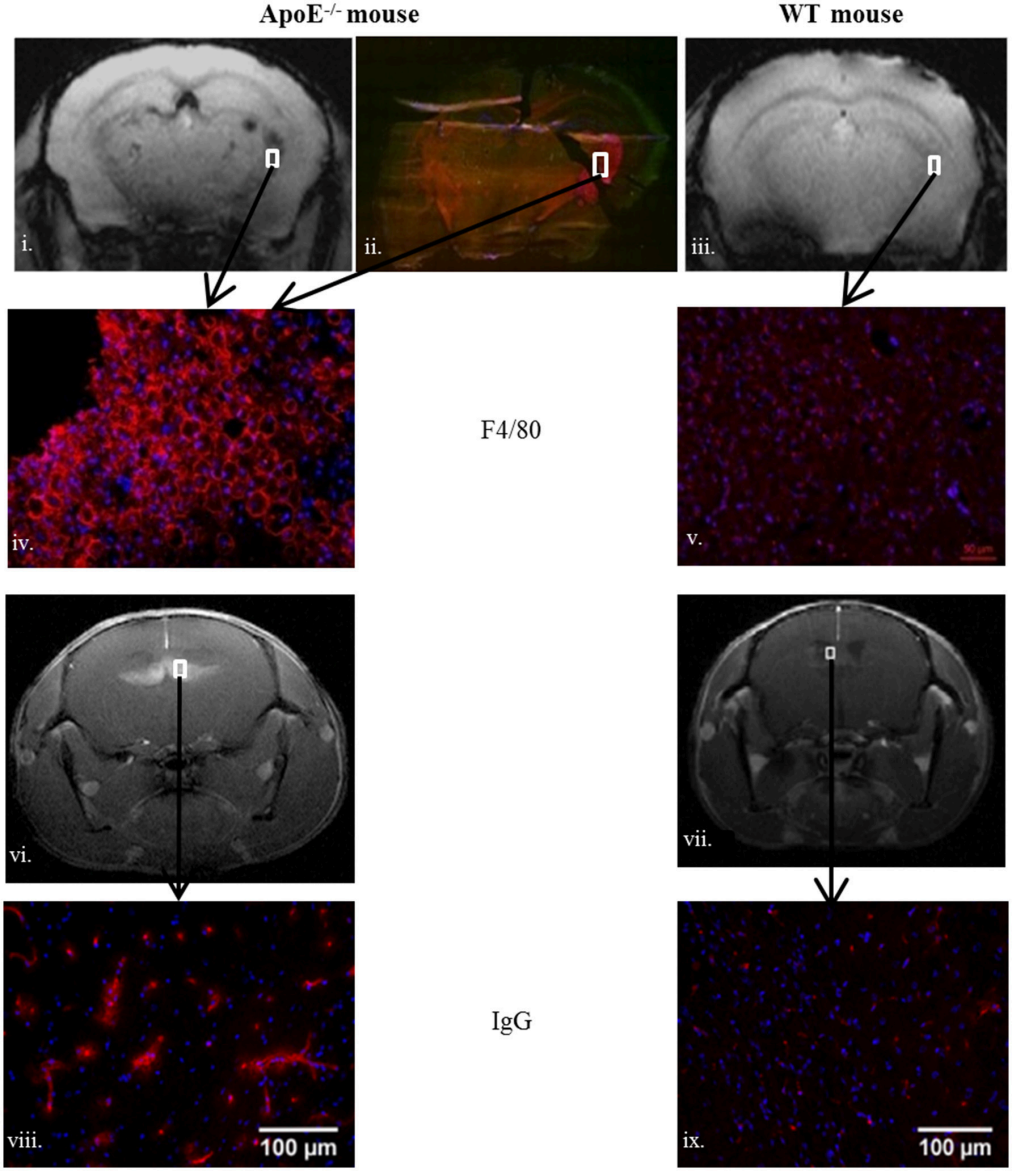
**Brain macrophage accumulation assessed by F4/80 (red color) in old ApoE^−/−^ (ii,iv) compared to sedentary old WT (v) and blood brain barrier leakage assessed by IgG (red color) in ApoE^−/−^ (viii) and WT (ix)**. White squares delineate the MRI corresponding periventricular zone in **(i,vi)** (ApoE^−/−^), **(iii,vii)** (WT). Areas of interest are fimbria (−2.06/−2.54 mm posterior to Bregma) for **(i,iii)** and septofimbrial nucleus and fornix (0/1.22 mm anterior to Bregma) for **(vi,vii)**. The ApoE^−/−^ mice MRI scores are: after-training post-Gd T1 score = 4 and after-training post-USPIOs T2 score = 2.5.

To characterize brain lesions in ApoE^−/−^ mice, WT mice served as negative control for the immunohistochemistry staining. In ApoE^−/−^ mice disorganized brain parenchyma was seen in the middle ventral zone, but was not related to an active apoptotic process (TUNEL negative on immunohistochemistry; data not shown). F4/80 and IgG immunostaining indicated BBB leakage (endothelial permeability), and macrophage accumulation in the periventricular region and the fornix fimbria of the hippocampus (Figure 4). Visually, there was also some evidence of vesicular aggregates which could indicate foam cell development and cholesterol crystals, as already described (Walker et al., 1997).

### Oxidative stress/inflammation in brain

**Figure 5:**
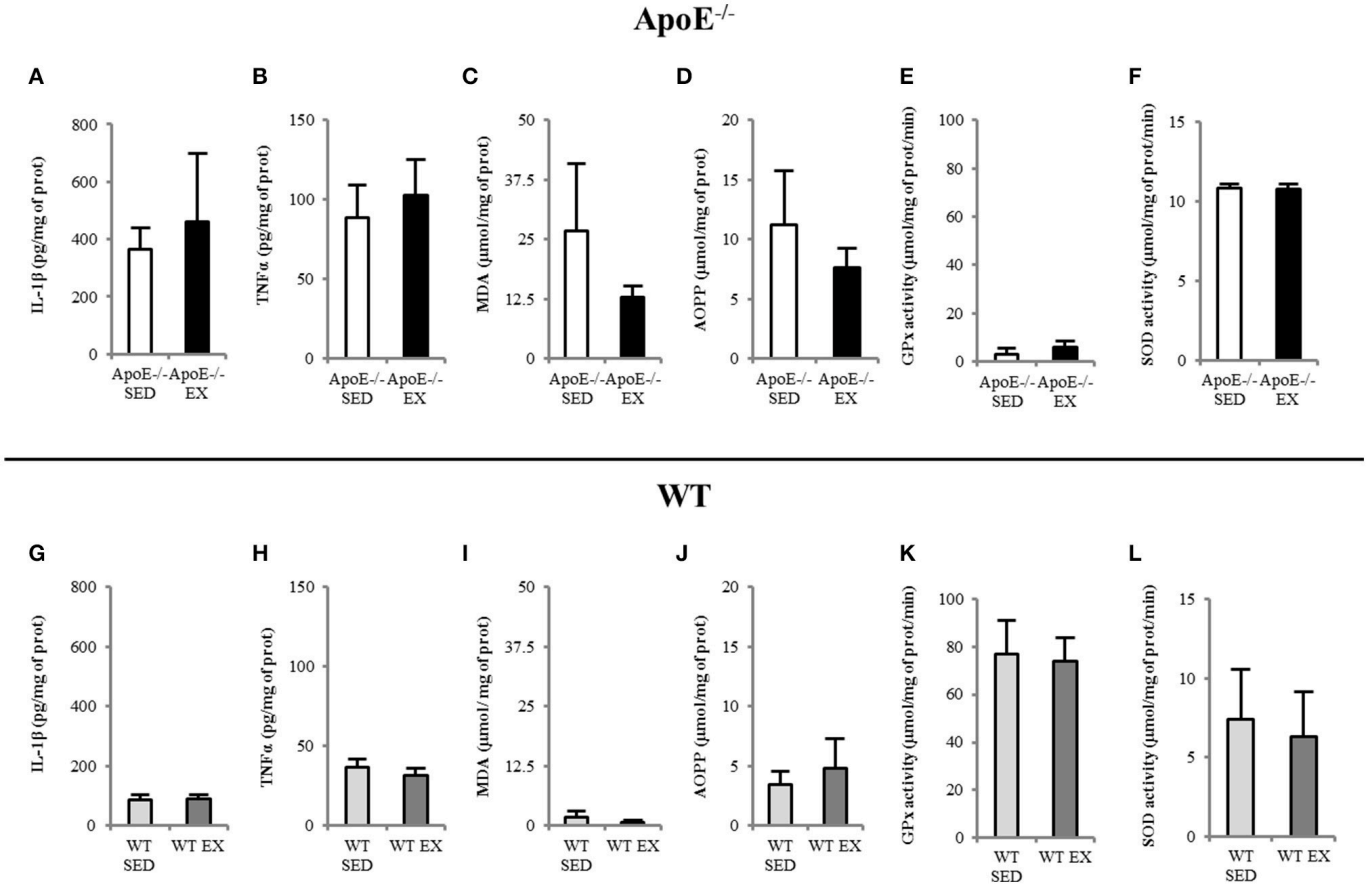
**EX and SED ApoE^−/−^ mice showed high brain inflammation level (A,B) and oxidative stress (C–F) in both SED and EX ApoE^−/−^ mice**. WT values are given for reference in brain inflammation **(G–H)** and oxidative stress **(I–L)**. IL-1β (Interleukin 1β) and TNFα (Tumor Necrosis Factor alpha), MDA (Malondialdehyde), AOPP (Advanced Oxidization Proteins Products), GPx (GluthationPeroxydase) activity, SOD (Superoxide Dismutase) activity.

Imaging data were confirmed by assays that showed that ApoE^−/−^ mice have high brain inflammation (IL-1β and TNFα; Figures 5A–B) and oxidative stress (MDA and AOPP were high, GPx had low activity; Figures 5C–E for ApoE^−/−^) regarding to reference values of WT mice (Figures 5G–L). No beneficial effect of exercise was noted in ApoE^−/−^ EX group.

### Oxidative stress/inflammation in plasma and aorta

In aorta, ApoE^−/−^ mice showed high concentration of the pro-inflammatory cytokines IL-1β and TNFα (Table S2). Oxidative stress was also present in ApoE^−/−^ mice as shown by the high AOPP level (protein oxidization; Table S2).

Plasmatic IL-1β was high in both EX and SED ApoE^−/−^ mice as well as oxidative stress (AOPP; Table S2). Reference values for WT mice are given in Table S2.

In these two tissues of interest, no beneficial effect of training was observed.

## Discussion

The aim of the present study was to determine both the peripheral and central effects of exercise training in 45 weeks-old ApoE^−/−^ mice fed *ad libitum* with a high fat/ high cholesterol (HC) diet using imaging and insulin tolerance test for follow-up. This diet given *ad libitum* was initially used to describe for the first time in the ApoE^−/−^ mouse the presence of vulnerable plaques, associated with a high mortality rate (Johnson and Jackson, 2001). We found that peripheral (insulin sensitivity and oxidative/inflammatory status) but also central features (BBB preservation and protection against inflammation) did not show any benefits of exercise. Indeed, there was a fast induction of irreversible brain damage that was more pronounced in exercise-trained ApoE^−/−^ mice.

While it is already known from large clinical studies that exercise training may encounter non or even adverse response for glucose homeostasis (Böhm et al., 2016), this study is the first to show that the combination of physical training and *ad libitum* HC diet also impairs central mechanisms in a mouse model of advanced atherosclerosis. Indeed, mature ApoE^−/−^ mice, i.e., with subsequent abnormal cholesterol handling, over-responded to the HC diet given *ad libitum*. *In vivo* MRI demonstrated the evolutive hippocampal consequences with an extremely fast induction of BBB leakiness that is correlated with an inflammatory burst with macrophage recruitment and high phagocytic activity (Figure 3). A parallel degradation of the systemic insulin response was measured (Figure 2). The observed consequences were neurological symptoms and mortality within the 12 weeks of follow-up (25 and 16% in EX and SED ApoE^−/−^, respectively). This clinical aggravation was observed despite a regular physical activity, which was supposed to induce protection and counteract neuroinflammation and oxidative stress, as previously described when the food quantity was limited to 20 g/week in both sedentary and exercise mice (Chirico et al., 2016). In this context of limited access to the HC diet, exercise training, possibly via its oxidative stress and inflammation lowering capabilities, reduced brain macrophage infiltration, limited inflammation, and oxidative stress in the brain, and also improved insulin sensitivity. In a recent study, 50-weeks old C57Bl/6 mice on *ad libitum* high fat diet presented peripheral and central metabolic consequences, but as these mice were not in a high cardiovascular risk, there was no pathological damage (Gotthardt et al., 2016). Therefore, this amplification in ApoE^−/−^ mice happened in a window where susceptibility to oxidative stress and hippocampal damage was high and was further aggravated by the abrupt HC diet exposure (HC diet starting 1 month before training), similar to what can be observed in the human transition phases such as retirement, where eating, and physical habits are particularly evolving whereas there is a parallel decrease of insulin sensitivity with age. A previous paper with diet-induced obese mice proposed a model where a high fat diet rich in saturated fat given *ad libitum* induced an excessive consumption food behavior associated with hippocampal dysfunction (Kanoski and Davidson, 2011). Here, the diet contained 21% lard and 0.15% cholesterol and was given without restriction, with more weight gain in EX ApoE^−/−^ mice compared to SED. Compared to other high cholesterol diets, this one is also richer in saturated fat (pork lard), which may have contributed to the insulin sensitivity degradation. In the early study of Johnson and Jackson, the high mortality rate was also attributed to this diet composition (Johnson and Jackson, 2001).

We also found that oxidative stress and inflammation were not decreased by exercise training in three tissues of interest (plasma, abdominal aorta, and brain). The measured markers presented similar (AOPP, SOD, and IL-1β) values in EX compared with SED ApoE^−/−^ mice. Insulin response and cholesterol level were also not different between these 2 groups although EX mice showed a significant higher citrate synthase activity in their soleus muscle, supporting the muscular enzymatic effect of physical training. In term of metabolism, there was no beneficial effect of training on either insulin resistance (as shown both in plasma and muscle, with even an aggravation of systemic insulin resistance in EX ApoE^−/−^ mice; Figure 2), or body composition (as assessed by the fat content measured by MRI) in old ApoE^−/−^ mice. The weight gain was even more pronounced in EX ApoE^−/−^ mice, and brain MRI clearly showed that ApoE^−/−^ EX mice were more affected both in terms of focal BBB leakage and of microglia/macrophage accumulation. All these features (lack of changes, even worsening of the pathological parameters) of the EX group, which are contradictory with the usual beneficial impact of exercise training (Xu et al., 2011; Rao et al., 2013), have been possibly driven by the weight gain observed in the EX ApoE^−/−^ mice when fed *ad libitum* with high HC diet.

In the present study, the ApoE^−/−^ mice under HC diet can be considered obese with regards to their weight at the beginning of the study (1.5-fold higher than age-matched WT mice). Furthermore, EX ApoE^−/−^mice also had a decreased response to insulin during the 12 weeks of training, suggesting that their insulin resistance state was worsened instead of being improved as commonly assumed from an endurance training protocol (Roberts et al., 2013). It is also interesting to note that abdominal fat was primary subcutaneous and visceral (Figure S1), as typically observed in metabolic syndrome patients or animal model (Patel and Abate, 2013). In obese subjects, it was observed that for the same physical activity level, those under food restriction presented reduced oxidative stress, inflammation and insulin resistance while those without food restriction experienced increased body weight and no improvement of fat distribution (Bergouignan et al., 1985). Similarly, in animal model, both inflammation and insulin sensitivity worsened in trained obese mice with *ad libitum* diet whereas it was fully improved when the diet was controlled (Ringseis et al., 2015).

Previous studies have shown that there are also specific locations for the positive effect of exercise on brain inflammation (Yi et al., 2012). However, in rodents, there seems to be major differences depending on the nature of the exercise (forced versus voluntary) in response to both the food intake control and the central effect of exercise (Leasure and Jones, 2008; Copes et al., 2015). Nevertheless, MRI studies on diet interventions have shown that diet-induced inflammation and gliosis are mainly located in the hypothalamic region (Thaler et al., 2012; Berkseth et al., 2014). Recently, in the context of obesity, the central effect of exercise to decrease neuroinflammation was described in the hippocampus region (Erion et al., 2014; Koga et al., 2014; Spielman et al., 2014; Auer et al., 2015).

An emerging field of research focuses on the new major roles of inflammation in brain diseases, where both its balanced deleterious and beneficial effects are still under debate (Mori et al., 2014). The complex interactions between the nervous system and the immune system are now thought to occur at specific interfaces such as the choroid plexus. Indeed, the brain anomalies that we observed in the old ApoE^−/−^ mice under HC diet are originally located in these areas and further extended from these periventricular areas (Leasure and Jones, 2008; Berkseth et al., 2014; Koga et al., 2014; Van der Donckt et al., 2015).

Due to the small number of animals, further studies would be needed to determine if there is any relationship between the activity level and brain inflammation. It would also be important to evaluate whether there is a difference of mortality rate between exercise and sedentary mice, or between male and female. Similar to the earlier study from Johnson and Jackson (2001), we did not observed any gender effect, but a larger animal group would be needed to further evaluate these possible differences.

Among the MRI biomarkers used in this study, the post-gadolinium T1 follow-up showed the unrevealed fast evolution of BBB leakiness in the ApoE^−/−^ mice after HC diet exposure. The T2 and T2^*^ scores were however unable to show a parallel evolution of tissue damage, which can be due to the small sample size. Yet, the change of T1 was strongly associated with the post-USPIO T2 score, and the two areas co-localized (Figure 4 and Figure S2). The evaluation of inflammation was done on T2 images (post-USPIO T2 score) rather than on T2^*^ maps because of technical issues. T2^*^ images are very sensitive to slight changes of MR signal homogeneities, such as those observed when surface coils are used for signal reception or after retro-orbital injection of USPIO. Yet, it is noticeable that periventricular inflammation developed around small existing focal T2^*^ lesions, as can be seen from T2^*^ maps (baseline and post-USPIO, Figure S2). These lesions can either be vascular microbleeds or brain accumulation of erythrocytes and iron deposit (Yi et al., 2012; Erion et al., 2014; Van der Donckt et al., 2015), as previously shown in this model (Chirico et al., 2016). Using a high magnetic field and a high-sensitivity transmit/receive volume radio-frequency coil, it was recently shown that higher spatial resolution T2^*^ mapping can detect very small quantity of iron or USPIO (Mori et al., 2014). In this specific study where neuro-inflammation was induced by a LPS challenge, there was no link between BBB leakage and inflammation. The new finding of rapid BBB leakage evolution can be compared to previous mechanistic studies where abnormalities of both aquaporin 4 and tight junction proteins expression were found in the same mouse model (Thaler et al., 2012). In apparent contradiction with the present result in this ApoE^−/−^ model, the destabilization of the neurovascular unit was partially modulated by exercise (Soto et al., 2015; Chirico et al., 2016). Yet, in our specific conditions of brutal HC diet exposure with no food consumption control, the imaging follow-up demonstrated aggravated BBB leakage that correlated with inflammation imaging, with high systemic, and brain inflammation and oxidative stress, and the brain status even worsened in exercise-trained mice. Future studies with higher spatial resolution MRI may provide deeper explanations of focal dynamic processes. An additional control group of ApoE^−/−^ mice under chow diet could also be included to follow the effect of exercise without diet intervention. As previously shown in ApoE^−/−^ mice under controlled diet intake (Chirico et al., 2016), they are likely to show a similar positive evolution if they have initial BBB lesion, or no evolution if they have normal BBB as our control group of age-matched WT mice.

In summary, we showed for the first time in this study that abrupt transition to HC diet with non-restricted consumption in old ApoE^−/−^ mice impairs the expected central beneficial effect of exercise, leading to enhanced inflammation and BBB leakage in the areas involved in immune cells recruitments. Therefore, this work emphasizes the need for further longitudinal studies in order to evaluate the neuro-immune interactions and elucidate the central effect of the interventions.

## Author contributions

VD, VP, and EC designed the study; VD, AG, JL, FC, BT, ENC, VP, and EC analyzed the data; VD, BT, FC, MW, HV, VP, and EC interpreted the data; VD, VP, and EC drafted the manuscript; VD, AG, FC, VH, MW, JR, HV, VP, and EC revised the manuscript critically for important intellectual content; VD, AG, JL, FC, BT, VH, MW, ENC, JR, HV, VP, and EC approved the final version of the manuscript submitted.

## Funding

This study was supported by the Institut Universitaire de France and the University Claude Bernard Lyon 1 for Ph. D. students.

### Conflict of interest statement

The authors declare that the research was conducted in the absence of any commercial or financial relationships that could be construed as a potential conflict of interest.

## Abbreviations

BBB: Blood-brain barrier
EX: Exercise-trained
HC: High cholesterol
MRI: Magnetic resonance imaging
SED: Sedentary
USPIO: Ultrasmall superparamagnetic particle of iron oxide
WT: Aged-matched C57Bl/6 Wild Type mice.
