# Supplementary material for: Exercise Does Not Protect against Peripheral and Central Effects of a High Cholesterol Diet Given Ad libitum in Old ApoE−/− Mice
Source: Front Physiol. 2016 Oct 6;7:453. doi: 10.3389/fphys.2016.00453 (PMC5052582; doi:10.3389/fphys.2016.00453)
Supplement: Supplementary file 1 [file Presentation1.PPTX]

## Slide 1
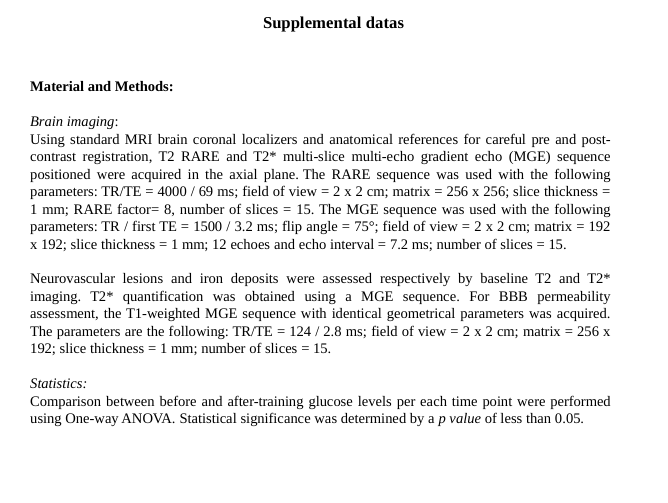

Supplemental datas
Material and Methods:
Brain imaging:
Using standard MRI brain coronal localizers and anatomical references for careful pre and post-contrast registration, T2 RARE and T2* multi-slice multi-echo gradient echo (MGE) sequence positioned were acquired in the axial plane. The RARE sequence was used with the following parameters: TR/TE = 4000 / 69 ms; field of view = 2 x 2 cm; matrix = 256 x 256; slice thickness = 1 mm; RARE factor= 8, number of slices = 15. The MGE sequence was used with the following parameters: TR / first TE = 1500 / 3.2 ms; flip angle = 75°; field of view = 2 x 2 cm; matrix = 192 x 192; slice thickness = 1 mm; 12 echoes and echo interval = 7.2 ms; number of slices = 15.
Neurovascular lesions and iron deposits were assessed respectively by baseline T2 and T2* imaging. T2* quantification was obtained using a MGE sequence. For BBB permeability assessment, the T1-weighted MGE sequence with identical geometrical parameters was acquired. The parameters are the following: TR/TE = 124 / 2.8 ms; field of view = 2 x 2 cm; matrix = 256 x 192; slice thickness = 1 mm; number of slices = 15.
Statistics:
Comparison between before and after-training glucose levels per each time point were performed using One-way ANOVA. Statistical significance was determined by a p value of less than 0.05.

## Slide 2
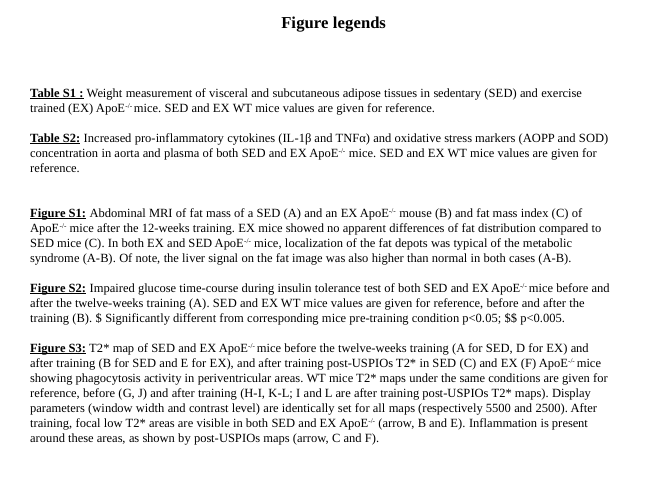

Figure legends
Table S1 : Weight measurement of visceral and subcutaneous adipose tissues in sedentary (SED) and exercise trained (EX) ApoE-/- mice. SED and EX WT mice values are given for reference.
Table S2: Increased pro-inflammatory cytokines (IL-1β and TNFα) and oxidative stress markers (AOPP and SOD) concentration in aorta and plasma of both SED and EX ApoE-/- mice. SED and EX WT mice values are given for reference.
Figure S1: Abdominal MRI of fat mass of a SED (A) and an EX ApoE-/- mouse (B) and fat mass index (C) of ApoE-/- mice after the 12-weeks training. EX mice showed no apparent differences of fat distribution compared to SED mice (C). In both EX and SED ApoE-/- mice, localization of the fat depots was typical of the metabolic syndrome (A-B). Of note, the liver signal on the fat image was also higher than normal in both cases (A-B).
Figure S2: Impaired glucose time-course during insulin tolerance test of both SED and EX ApoE-/- mice before and after the twelve-weeks training (A). SED and EX WT mice values are given for reference, before and after the training (B). $ Significantly different from corresponding mice pre-training condition p<0.05; $$ p<0.005.
Figure S3: T2* map of SED and EX ApoE-/- mice before the twelve-weeks training (A for SED, D for EX) and after training (B for SED and E for EX), and after training post-USPIOs T2* in SED (C) and EX (F) ApoE-/- mice showing phagocytosis activity in periventricular areas. WT mice T2* maps under the same conditions are given for reference, before (G, J) and after training (H-I, K-L; I and L are after training post-USPIOs T2* maps). Display parameters (window width and contrast level) are identically set for all maps (respectively 5500 and 2500). After training, focal low T2* areas are visible in both SED and EX ApoE-/- (arrow, B and E). Inflammation is present around these areas, as shown by post-USPIOs maps (arrow, C and F).

## Slide 3
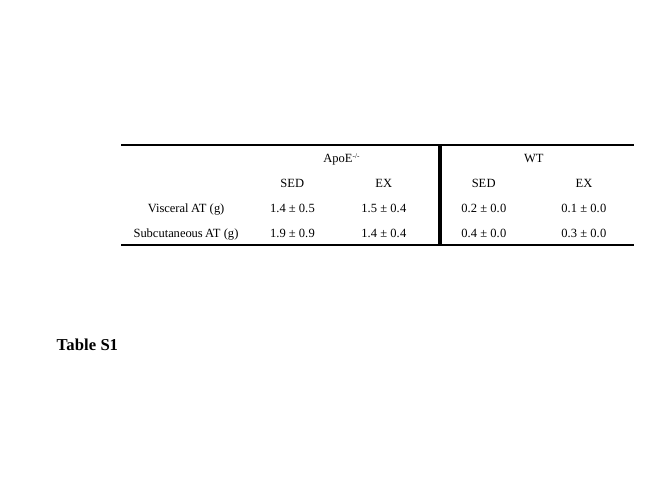

| | | ApoE-/- | | WT | |
| --- | --- | --- | --- | --- | --- |
| | | SED | EX | SED | EX |
| Visceral AT (g) | | 1.4 ± 0.5 | 1.5 ± 0.4 | 0.2 ± 0.0 | 0.1 ± 0.0 |
| Subcutaneous AT (g) | | 1.9 ± 0.9 | 1.4 ± 0.4 | 0.4 ± 0.0 | 0.3 ± 0.0 |
Table S1

## Slide 4
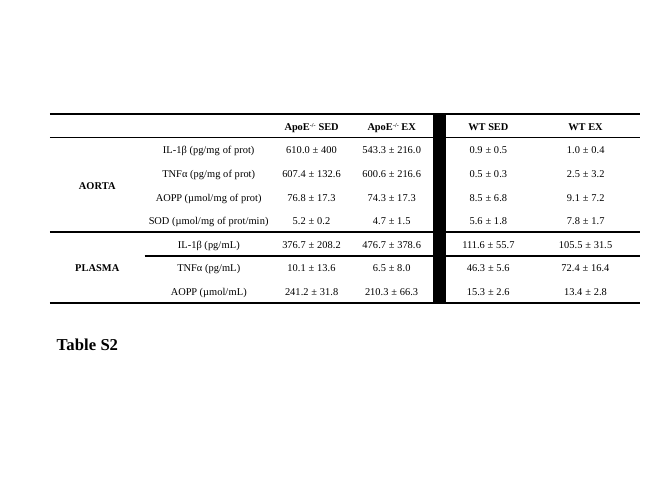

| | | ApoE-/- SED | ApoE-/- EX | | WT SED | WT EX |
| --- | --- | --- | --- | --- | --- | --- |
| AORTA | IL-1β (pg/mg of prot) | 610.0 ± 400 | 543.3 ± 216.0 | | 0.9 ± 0.5 | 1.0 ± 0.4 |
| | TNFα (pg/mg of prot) | 607.4 ± 132.6 | 600.6 ± 216.6 | | 0.5 ± 0.3 | 2.5 ± 3.2 |
| | AOPP (µmol/mg of prot) | 76.8 ± 17.3 | 74.3 ± 17.3 | | 8.5 ± 6.8 | 9.1 ± 7.2 |
| | SOD (µmol/mg of prot/min) | 5.2 ± 0.2 | 4.7 ± 1.5 | | 5.6 ± 1.8 | 7.8 ± 1.7 |
| PLASMA | IL-1β (pg/mL) | 376.7 ± 208.2 | 476.7 ± 378.6 | | 111.6 ± 55.7 | 105.5 ± 31.5 |
| | TNFα (pg/mL) | 10.1 ± 13.6 | 6.5 ± 8.0 | | 46.3 ± 5.6 | 72.4 ± 16.4 |
| | AOPP (µmol/mL) | 241.2 ± 31.8 | 210.3 ± 66.3 | | 15.3 ± 2.6 | 13.4 ± 2.8 |
Table S2

## Slide 5
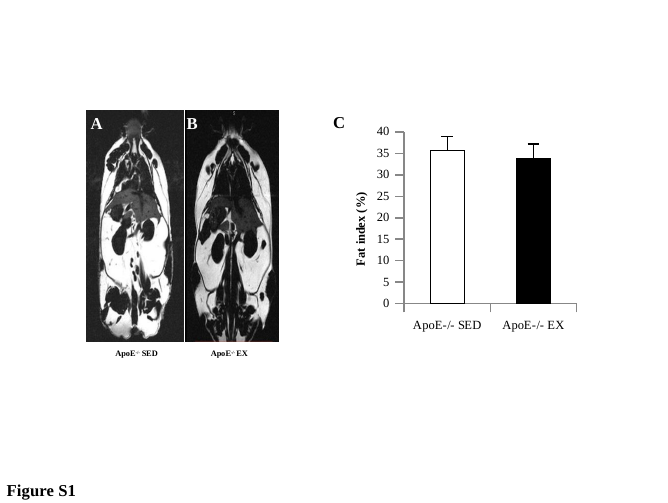

C
B
A
### Chart
| Category | |
|---|---|
| ApoE-/- SED | 35.7 |
| ApoE-/- EX | 33.85 |ApoE-/- SED
ApoE-/- EX
Figure S1

## Slide 6
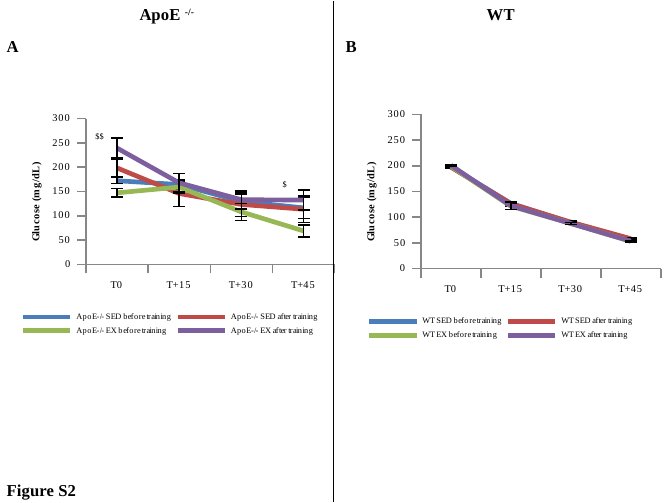

ApoE -/-
WT
A
B
### Chart
| Category | WT SED before training | WT SED after training | WT EX before training | WT EX after training |
|---|---|---|---|---|
| T0 | 197.85714285714286 | 197.57142857142858 | 199.33333333333334 | 201.16666666666666 |
| T+15 | 127.14285714285714 | 126.28571428571429 | 121.5 | 122.33333333333333 |
| T+30 | 89.57142857142857 | 90.57142857142857 | 88.0 | 87.83333333333333 |
| T+45 | 57.142857142857146 | 58.142857142857146 | 53.0 | 53.333333333333336 |
### Chart
| Category | ApoE-/- SED before training | ApoE-/- SED after training | ApoE-/- EX before training | ApoE-/- EX after training |
|---|---|---|---|---|
| T0 | 172.33333333333334 | 198.66666666666666 | 147.4 | 239.6 |
| T+15 | 164.5 | 146.0 | 159.2 | 168.0 |
| T+30 | 129.66666666666666 | 123.5 | 108.2 | 133.2 |
| T+45 | 116.5 | 113.66666666666667 | 69.0 | 132.6 |$$
$
Figure S2

## Slide 7
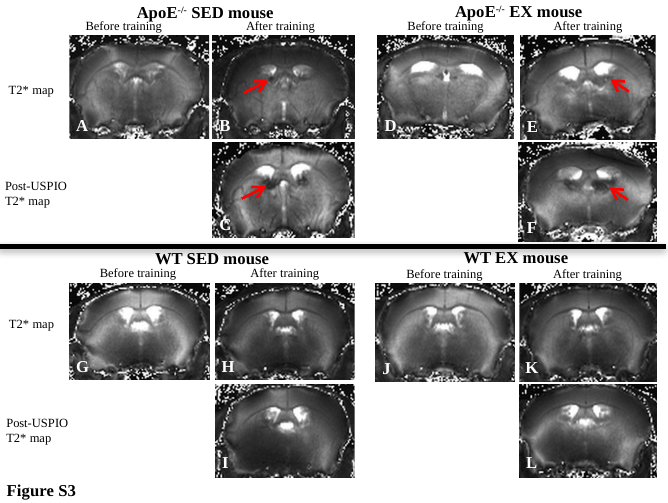

ApoE-/- EX mouse
ApoE-/- SED mouse
Before training
After training
Before training
After training
T2* map
A
B
D
E
Post-USPIO
T2* map
C
F
WT EX mouse
WT SED mouse
Before training
After training
Before training
After training
T2* map
H
G
K
J
Post-USPIO
T2* map
I
L
Figure S3
